# The Pyruvate Dehydrogenase Complex in Sepsis: Metabolic Regulation and Targeted Therapy

**DOI:** 10.3389/fnut.2021.783164

**Published:** 2021-12-14

**Authors:** Zhenhua Zeng, Qiaobing Huang, Liangfeng Mao, Jie Wu, Sheng An, Zhongqing Chen, Weijin Zhang

**Affiliations:** ^1^Department of Critical Care Medicine, Nanfang Hospital, Southern Medical University, Guangzhou, China; ^2^Department of Pathophysiology, Guangdong Provincial Key Lab of Shock and Microcirculation, Southern Medical University, Guangzhou, China; ^3^Department of Internal Medicine General Ward, Shantou Central Hospital, Shantou, China

**Keywords:** sepsis, glycolysis, metabolism, pyruvate dehydrogenase complex, therapy

## Abstract

Anaerobic glycolysis is the process by which glucose is broken down into pyruvate and lactate and is the primary metabolic pathway in sepsis. The pyruvate dehydrogenase complex (PDHC) is a multienzyme complex that serves as a critical hub in energy metabolism. Under aerobic conditions, pyruvate translocates to mitochondria, where it is oxidized into acetyl-CoA through the activation of PDHC, thereby accelerating aerobic oxidation. Both phosphorylation and acetylation affect PDHC activity and, consequently, the regulation of energy metabolism. The mechanisms underlying the protective effects of PDHC in sepsis involve the regulation on the balance of lactate, the release of inflammatory mediators, the remodeling of tricarboxylic acid (TCA) cycle, as well as on the improvement of lipid and energy metabolism. Therapeutic drugs that target PDHC activation for sepsis treatment include dichloroacetate, thiamine, amrinone, TNF-binding protein, and ciprofloxacin. In this review, we summarize the recent findings regarding the metabolic regulation of PDHC in sepsis and the therapies targeting PDHC for the treatment of this condition.

## Introduction

Sepsis is a major healthcare concern caused by an aberrant response to infection, which is easily complicated with multiple organ dysfunction syndromes (1–3). Despite the considerable progress for its clinical management, sepsis-related morbidity and mortality remain high (4), highlighting the importance of developing new strategies for sepsis prevention and treatment.

Sepsis is characterized by the release of a large number of inflammatory mediators, the imbalance in energy metabolism and the accumulation of lactic acid. The mechanisms underlying sepsis pathogenesis and development are complex, and involve inflammation, immunity, and metabolism, among other processes. Recent studies have shown that in the early stages of sepsis, high level inflammation relies on anaerobic glycolysis as the key energy source, while in the recovery stage, inflammation level was lowered and the cells switch to fat oxidation for their energy supply (5).

**Figure 1:**
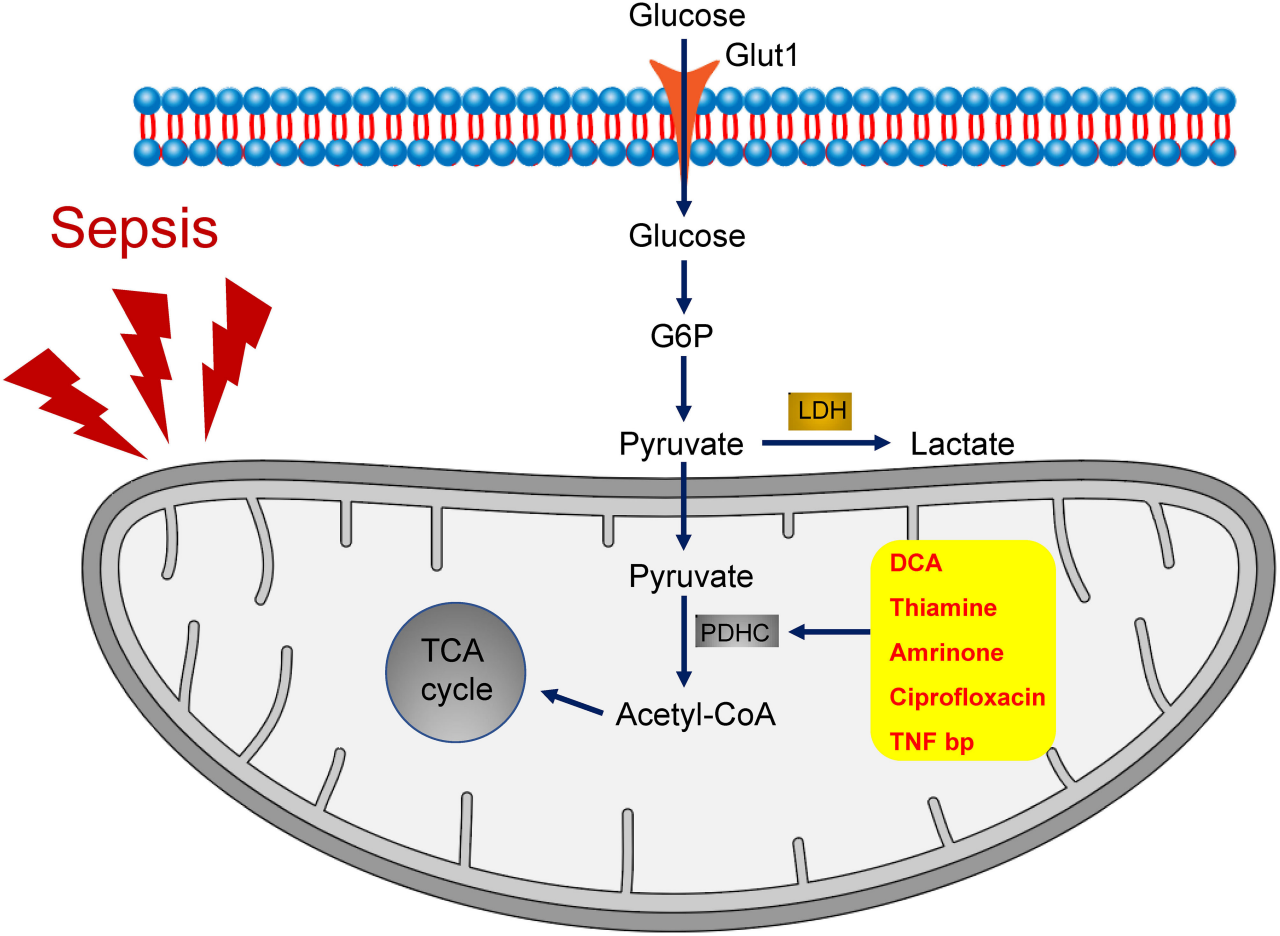
The role of PDHC in the metabolism of sepsis. During the process of glycolysis, one molecule of glucose is broken down into two molecules of pyruvate. Inactivation of PDHC results into anaerobic glycolysis, which is the primary metabolic pathway in sepsis. By contrast, activation of PDHC leads to pyruvate translocation to mitochondrial and the consequent acceleration of aerobic oxidation. A group of drugs that target PDHC activation, including dichloroacetate (DCA), thiamine, amrinone, ciprofloxacin, and TNF-binding protein (TNFbp), have been shown to ameliorate the symptoms of sepsis.

During the process of glycolysis, one molecule of glucose is broken down into two molecules of pyruvate. Under aerobic conditions, pyruvate translocates to mitochondria, where it is oxidized into acetyl-CoA through the activation of the pyruvate dehydrogenase complex (PDHC), thereby accelerating aerobic oxidation, whereas under hypoxic conditions, pyruvate produced through anaerobic glycolysis might result in the generation of lactic acid (Figure 1).

Although the amount of ATP produced by aerobic oxidation is significantly higher than that generated through anaerobic glycolysis, the speed of energy production *via* the former mechanism is significantly slower than that *via* the latter. Therefore, in the acute stage of sepsis, which is characterized by high energy consumption and hyper-inflammation, cells tend to generate energy to meet the significantly increased energy demands through anaerobic glycolysis, during which pyruvate converts into lactate. Furthermore, an absence of acetyl-CoA generated *via* pyruvate oxidation remodeled the tricarboxylic acid (TCA) cycle and lipid metabolism, with accumulation of two intermediates which could be produced through other pathways other than the physiological TCA cycle upon sepsis, namely succinate and citrate, both of which are pro-inflammatory mediators. In macrophages treated with lipopolysaccharide (LPS), succinate could be produced through anaplerosis of α-ketoglutarate into the TCA cycle and the subsequent increased glutamine metabolism. Moreover, LPS also increases γ-aminobutyric acid and its transporters, thus producing succinate. Citrate accumulates due to the shunting of cis-aconitate to itaconate through the enzymatic action of aconitase decarboxylase. Furthermore, free fatty acids and glycerol are released abundantly from adipose tissue into bloodstream, and are taken up by the liver to be oxidized into β-oxidation with the increase of acetyl-CoA, to produce more energy and ketone bodies, used as the source of energy by organs. However, this process is accompanied by the increased levels of palmitic acid and palmitoyl-carnitine, which might induce mitochondrial dysfunction. Together, lipotoxicity is also induced, characterized by the accumulation of malondialdehyde and 4-hydroxynonenal, which are the end products of lipid radical reactions and could cause cell death in liver and kidney (6–9). Following the acute stage of sepsis is the hypo-metabolic stage, which is characterized by low energy demand and hypo-inflammation. Cells are reprogrammed to low-energy state through aerobic oxidation, with the conversion of pyruvate to acetyl-CoA and the appropriate procession of TCA cycle and lipid metabolism (10). Switching metabolism to adjust energy supply and modulate inflammation in response to different stresses, including sepsis, is known as metabolic reprogramming. Early metabolic reprogramming in the acute phase of sepsis may be detrimental (11). Furthermore, early metabolic reprogramming in sepsis markedly alters lipid metabolism, resulting in lipotoxicity and glycerol accumulation (7, 9).

The generation of lactic acid, the major byproduct of anaerobic respiration, is the most frequently observed metabolic consequence of sepsis. Lactic acid can, in turn, activate Toll-like receptor 4 (TLR4) and promote the activation of nuclear factor-kappa B (NF-κB), as well as the further release of inflammatory mediators (12). Studies have shown that reducing lactic acid levels in the early stage of sepsis can improve the prognosis of patients (13, 14), suggesting that inhibiting early metabolic reprogramming, and subsequently decreasing subsequent lactate production and metabolic impairment, may represent a promising target for preventing the development of sepsis.

However, switching between these two metabolic pathways is necessary and might have beneficial effects. It is reported that, during the early stages of sepsis, metabolism in tubular epithelial cells can switch from aerobic oxidation to anaerobic glycolysis, suggestive of metabolic reprogramming (15). This alteration might enhance the capacity of cells to eliminate mitochondrial reactive oxygen species (ROS) accumulated due to aerobic oxidation in sepsis (16). Anaerobic glycolysis can also promote the pentose phosphate pathway and the production of NADPH, thus regenerating reduced glutathione, which favors the elimination of hydrogen peroxide (17). During sepsis, metabolic reprogramming helps to downregulate major energy sinks such as ion transport and fuel processes that are necessary for cell survival, thus contributing to energy maintenance (18). Furthermore, this switch from aerobic oxidation to anaerobic glycolysis is beneficial for the development of trained immunity, which helps build up the innate immune system to defend against future stresses (16). This study also demonstrated that a shift from oxidative phosphorylation to anaerobic glycolysis in glucose metabolism is the metabolic basis for trained immunity (19). In this context, early metabolic reprogramming and energy prioritization during sepsis might alleviate the extent of organ dysfunction, the progression to fibrosis, and the development of chronic kidney disease (16).

Concerning the wide-ranging effects of metabolic reprogramming, both beneficial and detrimental, further work is warranted for an improved understanding of the role of the different metabolic pathways in sepsis, as well as identifying strategies to improve outcome in septic patients through the regulation of metabolism.

A large number of studies have focused on PDHC, the key regulator of glucose metabolism, in exploring the underlying pathogenesis of sepsis (20). Accordingly, PDHC has been shown to regulate critical processes, including lactate production, the release of inflammatory mediators, TCA cycle, lipid metabolism and energy production. Here, we provide an overview of recent findings relating to how PDHC regulates metabolism in sepsis.

## Function and Regulation of PDHC

PDHC, a multienzyme complex composed of pyruvate dehydrogenase E1 (PDHE1), dihydrolipoamide transacetylase E2 (PDHE2), and dihydrolipoamide dehydrogenase E3 (PDHE3), serves as the critical hub in energy metabolism. Numerous studies have shown the positive regulatory role of PDHC in anaerobic glycolysis in sepsis. One study showed that PDHC activity was decreased by ~70% in skeletal muscle cells of septic rats, leading to cellular hypoxia and dysfunction (21). In terms of clinical research, Nuzzo et al. demonstrated that PDHC activity was significantly lower in peripheral blood mononuclear cells from sepsis patients than in those of healthy controls, and that this reduced activity may affect the prognosis of patients with sepsis (22). This suggests that PDHC activity may serve as a critical marker for the pathogenesis and development of sepsis.

PDHC is regulated *via* multiple mechanisms, including phosphorylation and acetylation. PDH kinase (PDK), consisting of four isoforms (PDK1–PDK4), acts as an upstream negative regulator of PDHC and is located in the mitochondrial matrix (23). PDK can decrease PDHC activity through the phosphorylation of PDHE1α on Ser293, Ser300, and Ser232, thereby inhibiting the TCA cycle and resulting in the accumulation of pyruvate and the production of lactic acid. In contrast, PDHC activity can be restored *via* PDH phosphatase (PDP)-mediated dephosphorylation (24). In skeletal muscle, the levels of the non-phosphorylated form of PDH and PDHC activity are both reduced following the induction of sepsis. Meanwhile, PDHC phosphorylation was reported to result in hyperlactatemia and disorder in energy metabolism (21). A different study showed that in LPS-stimulated mouse digital extensor muscle cells, the PDK mRNA expression level was upregulated 24-fold, while PDHC activity was decreased by 65%, resulting in an increase in lactic acid levels (25). These findings indicate that PDK is an important regulator of energy metabolism through its capacity to phosphorylate and inactivate PDHC.

Besides phosphorylation, studies have shown that PDHC acetylation also affects its activity and exerts a regulatory effect on energy metabolism (26). Notably, the acetylation level of PDHC is upregulated by acetyl-CoA acetyltransferase 1 (ACAT1), while PDHC deacetylation is catalyzed by the deacetylase SIRT3 (27). In skeletal muscle, an increase in PDHE1α acetylation levels reduces the activity of PDHC and causes metabolic disorder. That the switch from aerobic to anaerobic oxidation likely results in lactic acid accumulation indicates that PDHE1α acetylation plays a significant role in the regulation of PDHC activity and energy metabolism (28). Recent studies have also confirmed that increased levels of PDHC acetylation can lead to reductions in PDHC activity and total ATP synthesis, leading to mitochondrial dysfunction and myocardial damage (29). Consistent with these results, PDHE3 acetylation levels were sharply increased in a model of myocardial metabolic remodeling in obese mice, supporting the pivotal role of PDHE3 acetylation in mediating obesity-induced myocardial injury (30). However, relatively few studies have investigated the mechanism underlying the role of PDHC acetylation in sepsis.

**Figure 2:**
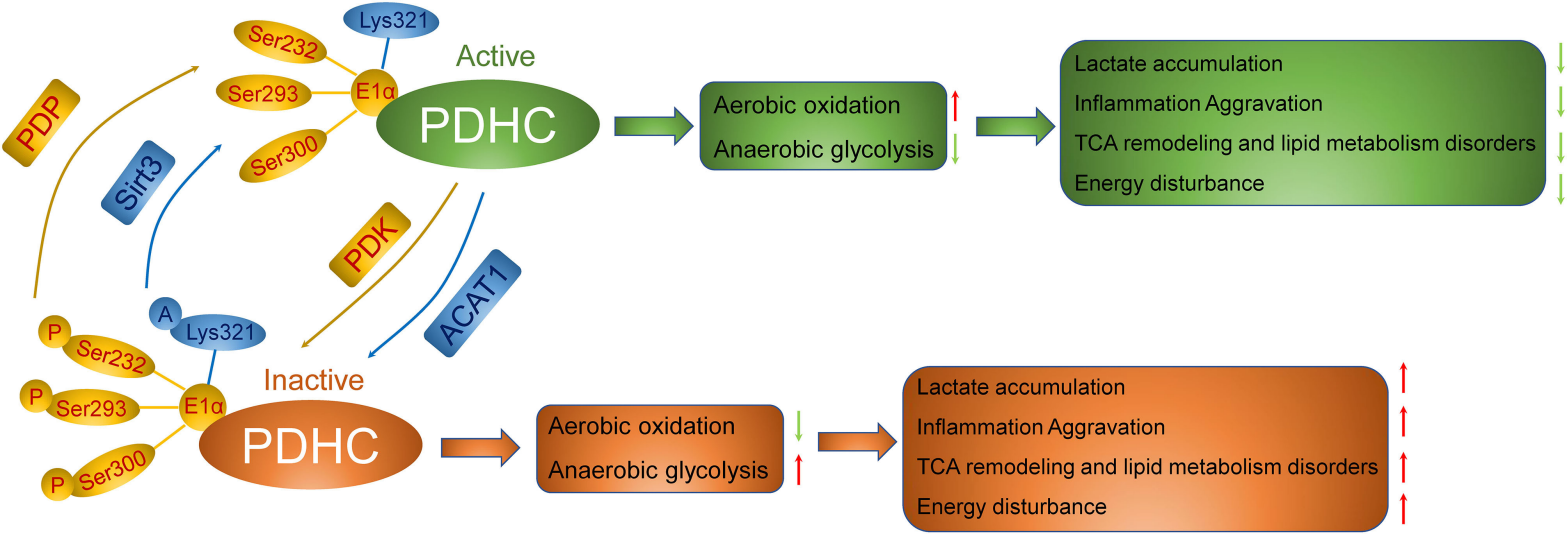
Regulation of PDHC in the metabolism of sepsis. The inactivation of PDHC through PDK-mediated phosphorylation and acetylation by ACAT1 results into anaerobic glycolysis, thereby inducing accumulation of lactic acid, the aggravation of inflammation, the remodeling of TCA cycle, the disorder of lipid metabolism, and the consequence of lower ATP synthesis relative to ATP demand. Activation of PDHC instead, *via* PDP-mediated de-phosphorylation and de-acetylation by Sirt3, leads to aerobic glycolysis, which is good for amelioration of sepsis.

Furthermore, there is crosstalk between the phosphorylation and acetylation of PDHC. The enhancement of PDHC acetylation through ACAT1 activation helps recruit PDK to the phosphorylation sites of PDH, thus inducing a decrease in PDHC activity, while the blockade of PDHC acetylation through SIRT3 activation may lead to its dephosphorylation and activation (31). Together, these results reveal that the direct targeting of PDHC acetylation or phosphorylation may be a promising strategy for sepsis management (Figure 2).

## Pathological Effect Caused by PDH Imbalance

As a critical node in metabolic regulation, PDHC converts pyruvate to acetyl-CoA in mitochondria, and its inhibition leads to early metabolic reprogramming in response to sepsis, resulting in the accumulation of lactic acid, the aggravation of inflammation, the remodeling of TCA cycle, and the disorder of lipid metabolism, and the consequence of lower ATP synthesis relative to ATP demand.

### Lactate Accumulation

The inhibition of PDHC activation and the switch from aerobic oxidation to anaerobic glycolysis in sepsis is known to contribute to the formation of a large amount of lactic acid and intracellular acidosis, which can lead to intracellular Ca^2+^ overload, mitochondrial membrane damage (32, 33). The persistence of hyperlactatemia is responsible for arrhythmia, kidney injury, respiratory failure, central nervous system dysfunction, and damage to several other organs (34). There is evidence supporting that lactate triggers innate immune responses via TLR-mediated enhancement of NF-κB transcriptional activity and subsequent expression of pro-inflammatory genes in macrophages (12). Moreover, lactate has also been reported to promote HMGB1 acetylation in macrophages (35). From a clinical perspective, reducing lactic acid levels in the early stage of sepsis has been reported to improve the prognosis of patients (36). Collectively, these findings shed light on how inflammation and mitochondrial injury might be controlled through the regulation of lactate release in the treatment of sepsis.

As mentioned above, PDHC is a critical regulator of lactate levels. Bakalov et al. (20) confirmed that sepsis could lead to decreased PDHC activity and increased lactic acid levels in the gross tissues of Drosophila, while the upregulation of PDHC activity could reduce lactic acid levels and improve the survival rate of the flies. In a rat model of sepsis, inhibiting PDHC activity can lead to a significant increase in the level of lactate, illustrating the negative regulatory effect exerted by PDHC activity on the level of lactic acid (37). In addition, other studies have found that in septic mice, the administration of PDHC agonists can reduce the levels of lactate, thereby improving the disrupted metabolic regulation in the animals (38). These observations suggest that PDHC-mediated changes in lactate levels play a key role in sepsis-related metabolic changes. Accordingly, levels of PDHC activity and lactic acid may serve as biomarkers for the occurrence of sepsis.

### Aggravation of Inflammation

Anaerobic glycolysis promotes the activation of a variety of inflammatory cells, including monocytes, dendritic cells, and macrophages (39, 40). Lactate, as a pro-inflammatory metabolite that could regulate macrophage polarization and increase the production of pro-inflammatory factors, is a marker for sepsis severity (41). Lactate has also been demonstrated to promote the expression of vascular endothelial growth factor, while lactate inhibition could block TLR4 signaling and attenuate the production of pro-inflammatory factors (12). Given that lactate is the major product of PDHC inactivation in anaerobic glycolysis, the targeting of PDHC may be a means for controlling lactate production and downstream inflammation pathways in sepsis.

Studies have confirmed that PDK is involved in the regulation of macrophage differentiation and glucose metabolism in bone marrow macrophages of septic mice. PDK1 knockout reduced the phosphorylation level of PDHE1α and upregulated the activity of PDHC, thus inhibiting LPS-induced anaerobic glycolysis and the polarization of macrophages from an M2 to an M1 phenotype and, consequently, reducing the levels of the inflammatory mediators interleukin (IL)-6, IL-12, and IL-1β. This finding highlights that PDK inhibition exerts positive regulatory effects in glycolysis *via* PDHC, allowing M1 macrophages to be polarized toward the M2 phenotype, thus tempering the release of inflammatory mediators (42). Bakalov et al. showed that PDHC activation led to the lowered level of cecropin-A and defensin, two releasing markers of pro-inflammatory factors, resulting in the improvement of life span of septic Drosophila (20). These results indicate that regulation of PDHC activity may represent a therapeutic target in the treatment of sepsis.

### TCA Remodeling and Lipid Metabolism Disorders

It is reported that in hepatocytes under septic condition, multiple TCA cycle-related metabolites, including citrate, cis-aconitate, and succinate, were markedly upregulated, indicating the remodeling of TCA (7). Citrate, exported from mitochondria *via* the mitochondrial citrate carrier (CIC), is a key molecule for the generation of energy. After citrate synthesis in the mitochondrial, citrate can enter the Krebs cycle and promote oxidative phosphorylation for energy production, which is required for the production of inflammatory molecules after LPS treatment. In the cytoplasm citrate is cleaved to acetyl-CoA which is the precursor for fatty acid, or cleaved to oxaloacetate which is converted to malate and then pyruvate (43). Excessive succinate accumulation following TCA remodeling in sepsis primes inflammation through succinate dehydrogenase (SDH)-mediated ROS generation and IL-1β production (44). In addition to TCA remodeling, lipid metabolism is also reprogrammed in sepsis. Studies showed that the amount of free fatty acids from the white adipose tissue into bloodstream was elevated under infection (45). Lipidomic analysis in livers of septic mice also demonstrated fatty acid uptake and β-oxidation are upregulated in sepsis to produce ketone bodies used as an energy source by the brain and other bodies, leading to excess free fatty acids and triglycerides, causing lipotoxicity (7, 9). Moreover, the hepatic levels of phospholipids, including phosphatidylcholine, phosphatidylethanolamine, and sphingomyelin, were also significantly elevated, all of which influence energy metabolism and have been linked with sepsis progression (46).

Given its critical role in the regulation of the TCA cycle and lipid metabolism, the impairment of which underlies the organ dysfunction observed in sepsis, PDHC may represent an effective target for sepsis treatment. The activation of PDHC significantly restored TCA metabolite levels to those of control and improved liver function in sepsis (7). PDHC activation also restored anabolic energy in inflammatory monocytes while also increasing the abundance of TCA cycle intermediates and the anaplerotic metabolism of branched-chain amino acids, thus promoting TCA-driven anabolic energetics (47). Furthermore, PDHC activation in septic mice reversed lipid disorder and mitochondrial dysfunction, indicative of the positive regulatory role of PDHC in lipid metabolism. Combined, these observations demonstrate that PDHC is a critical regulator of TCA cycling and lipid metabolism during sepsis.

### Energy Disturbance

PDHC is essential for glucose oxidation through its ability to promote a switch from the glycolytic to the oxidative pathway and the subsequent use of substrates through the respiratory chain in mitochondria. PDHC catalyzes the oxidative decarboxylation of pyruvate, yielding NADH and acetyl-CoA, key molecules for mitochondrial ATP generation (48). During sepsis, the downregulation of PDHC might contribute to energy metabolism disturbances by impairing the capacity of mitochondria for energy production. Furthermore, PDHC inactivation might help to explain the lower functional capacity of mitochondrial energy in premature neonates with sepsis compared with older children patients (49). Studies have shown that PDHC activation induced by norepinephrine could improve the respiratory function in mitochondria and attenuate the inflammation in septic rats (50). These data all support that enhancing PDHC activity and maintaining metabolism homeostasis can restore mitochondrial function and inhibit organ dysfunction in sepsis.

## PDHC Activators for the Improvement of Sepsis

Given its critical role in the metabolism of sepsis, PDHC may represent an effective target for sepsis management. A study by McCall et al. (51) confirmed that the promotion of PDHC activity through PDK inhibition facilitates immune-metabolic adaptations in sepsis. A group of drugs that target PDHC activation, including dichloroacetate (DCA), thiamine, amrinone, ciprofloxacin, and TNF-binding protein (TNFbp), have been shown to ameliorate the symptoms of sepsis (Figure 1).

### DCA

DCA is a small-molecule metabolism-regulating drug that is mainly used in the treatment of diseases related to mitochondrial defects and lactic acid accumulation (52). It is well-known as a PDK inhibitor and can reduce the level of PDHC phosphorylation by inhibiting the activity of PDK, upregulating the activity of PDHC, and promoting the entry of pyruvate into mitochondria for oxidative phosphorylation. Several studies have confirmed that DCA can activate PDHC, regulate glucose metabolism, and inhibit lactic acid accumulation in septic cells (53). The activation of PDHC with DCA significantly restored TCA metabolite levels to those of control and improved liver function in sepsis (7).

Furthermore, DCA administration to septic mice reversed lipid disorder and mitochondrial dysfunction, indicative of the positive regulatory role of PDHC in lipid metabolism. Similarly, compared with control septic animals, the activation of PDHC through DCA infusion led to decreases in plasma lactate concentrations and glycolytic activity, thus restoring normal glycolytic function (38).

Notably, clinical studies have also shown that DCA can promote a switch in glucose metabolism from anaerobic glycolysis to oxidative phosphorylation in patients with sepsis, thereby significantly inhibiting the occurrence of hyperlactatemia (54). These results suggest that DCA can effectively improve prognosis in septic patients. However, one randomized controlled study reported that DCA injection could significantly reduce lactate levels in sepsis patients, but did not significantly affect hemodynamics or mortality (55). Additionally, DCA has been associated with several side effects (56), highlighting that the clinical application of DCA requires further extensive verification.

### Thiamine

Thiamine, also known as vitamin B1, consists of pyrimidine and thiazole moieties, and thiamine deficiency can lead to the disease beriberi (57). Studies have shown that thiamine, a coenzyme of PDHC, plays an important role in improving the activity of the latter in sepsis. Between 10 and 30% of patients with critically illness are deficient in thiamine (58). For patients with sepsis, thiamine deficiency can lead to the accumulation of pyruvate and induce the production of a large amount of lactate, thus significantly increasing the mortality of patients (59). Injecting thiamine is beneficial for mitigating increases in lactate levels and can also improve prognosis in sepsis patients (60, 61). Given the potential health-related significance of thiamine, current guidelines recommend that patients admitted to ICU should receive 100–300 mg of thiamine daily for the first 3 days to reduce the potential adverse prognosis of sepsis (62). Important though thiamine is in clinical drug application, further studies are required for thiamine dose determination and for identifying the causal relationship between PDHC activity regulation and the clinical effect of thiamine.

### Other Activators

Amrinone, which was reported to inhibit TNF synthesis, could prevent alterations in muscle protein metabolism produced by sepsis. Burns et al. (63) found that in rats with septic shock, amrinone could induce a 2.5-fold increase in PDHC activity in cardiac tissue compared with that of control animals, and also increased the ATP level. Additionally, it has been shown that the injection of amrinone at a dose of 5 mg/(kg·day^−1^) for 5 days in septic rats helped to induce the activation of PDHC in skeletal muscle and to significantly reduce the level of lactate, suggesting that amrinone may be useful for the treatment of hyperlactatemia in septic patients (64). However, further evidence-based data are needed before amrinone can be administered clinically to septic patients.

Ciprofloxacin, a member of the quinolone family, can downregulate the levels of PDK1, which, in turn, activates PDHC, reverses the loss of ATP, and decreases the high mortality of mice exposed to ionizing radiation and trauma (65). It is imperative to determine whether ciprofloxacin provides therapeutic benefit for sepsis.

Studies have demonstrated that TNFbp injection can increase the activity of PDHC in skeletal muscle cells as well as reverse the increase in lactate levels in septic rats. Investigation of the effects of TNFbp in sepsis is ongoing (37).

## Conclusion

Most studies to date have focused on the role of PDHC phosphorylation in sepsis, while reports on the effect of acetylation and the crosstalk between these two post-translational modifications are scarce. While we summarized the mechanisms underlying the role of PDHC in lactate production, inflammation, TCA cycle, and in lipid and energy metabolism (Table 1), whether other mechanisms are also involved remains to be determined. Regarding to the targeting treatment, although several drugs have been reported to improve the prognosis of patients with sepsis by activating PDHC, evidence-based data are still lacking. Many unsolved problem remain. Further exploration of the role of PDHC in sepsis is required for the understanding of septic pathogenesis and the management of sepsis.

**Table 1:** Overview of included studies.

**Reference**	**Research object**	**Intervention**	**PDHC activity change**	**Major effect on metabolism in sepsis**
Mainali et al. (7)	Hepatocytes of male C57BL/6J	Dichloroacetate	**↑**	Dysregulated hepatocyte metabolism and mitochondrial dysfunction were reversed
Bakalov et al. (20)	Drosophila	Dichloroacetate	**↑**	Normalized lactate and TCA metabolites, and improved lifespan
Vary (21)	Hindlimb skeletal muscle of male Sprague-Dawley rats	Escherichia coli plus bacteroides fragilis	**↓**	Sustained hyperlactatemia
Vary et al. (37)	Hindlimb skeletal muscle of male Sprague-Dawley rats	TNF binding protein	**↑**	Hyperlactatemia were prevented
L'Her and Sebert (38)	Blood from the internal jugular vein and lateral gastrocnemius muscle of male Sprague-Dawley rats	Dichloroacetate	**↑**	Lactate content was decreased and glucose content was increased
Tan et al. (42)	Bone marrow-derived macrophages from C57BL/6 male mice	PDK1 siRNA	**↑**	M1 was diminished, whereas M2 activation and mitochondrial respiration was enhanced
McCall et al. (51)	Splenocyte and hepatocyte from C57BL/6 male mice	Dichloroacetate	**↑**	Mitochondrial oxidative bioenergetics was increased, vascular and organ homeostasis was promoted, and survival rate was increased
Giacalone et al. (60)	Three patients with severe lactic acidosis	Thiamine	**↑**	A rapid and marked restoration of acid-base balance
Burns et al. (63)	Hearts from adult male Sprague-Dawley rats	Amrinone or dichloroacetate	**↑**	Myocardial ATP levels were elevated, and myocardial oxidation of glucose was enhanced
Vary (64)	Hindlimb skeletal muscle of male Sprague-Dawley rats	Amrinone	**↑**	Reduced lactate concentrations

## Author Contributions

ZZ and QH contributed to the organization and writing of the manuscript. LM, JW, SA, and ZC wrote sections of the manuscript. WZ reviewed and edited the submitted version. All authors approved the manuscript for publication.

## Funding

This work was supported by grants from National Natural Science Foundation of China (Grants 81900279).

## Conflict of Interest

The authors declare that the research was conducted in the absence of any commercial or financial relationships that could be construed as a potential conflict of interest.

## Publisher's Note

All claims expressed in this article are solely those of the authors and do not necessarily represent those of their affiliated organizations, or those of the publisher, the editors and the reviewers. Any product that may be evaluated in this article, or claim that may be made by its manufacturer, is not guaranteed or endorsed by the publisher.
